# Short-Term Outcomes of Partial Upper Ministernotomy for Aortic Valve Replacement Within the Learning Curve Context

**DOI:** 10.3390/jcdd12070254

**Published:** 2025-07-01

**Authors:** Tomáš Toporcer, Marián Homola, Anton Bereš, Michal Trebišovský, Tomáš Lopuchovský, Štefánia Mižáková, Lukáš Vajda, Štefan Lukačín, Adrián Kolesár

**Affiliations:** Department of Heart Surgery, East Slovak Institute for Cardiovascular Diseases and Medical Faculty, Pavol Jozef Šafárik University, 04011 Košice, Slovakia; topyto@gmail.com (T.T.); aberes@vusch.sk (A.B.); mtrebisovsky@vusch.sk (M.T.); tlopuchovsky@vusch.sk (T.L.); smizakova@vusch.sk (Š.M.); lvajda@vusch.sk (L.V.); slukacin@vusch.sk (Š.L.); akolesar@vusch.sk (A.K.)

**Keywords:** partial upper mini-sternotomy, aortic valve replacement, aortic valve stenosis

## Abstract

Background: In recent decades, aortic valve surgery has transitioned from conventional median sternotomy (MS) to minimally invasive techniques, including partial upper mini-sternotomy (PUMS) and right anterolateral mini-thoracotomy (RAMT). This study retrospectively compares the outcomes of aortic valve replacement (AVR) using PUMS during the learning phase with those of standard MS. Methods: A retrospective analysis was conducted on patients (n = 211) who underwent AVR for aortic stenosis. They were divided into MS (n = 119) and PUMS (n = 92) groups. Various preoperative, surgical and postoperative parameters, including survival, were examined. Results: Preoperatively, the main difference was age, with PUMS patients being older (67.5 ± 7 vs. 66.5 ± 9.6; *p* = 0.010). PUMS patients also had longer cardiopulmonary bypass (CPB) and cross-clamping times (99 ± 25 vs. 80 ± 16 min; *p* < 0.002; 79 ± 18 vs. 65 ± 13 min; *p* < 0.024). There were no significant differences in body mass index, prosthesis size, indexed effective orifice area, hospitalisation duration or any other monitored parameter. Echocardiographic follow-up found no differences in prosthetic pressure gradients, flow velocity or paravalvular leak between the PUMS and MS groups. Survival rates were similar over 1000 days. Conclusions: The data suggest that PUMS offers comparable surgical outcomes to MS for AVR with additional cosmetic benefits, undeterred by a learning curve.

## 1. Introduction

Aortic stenosis (AS) represents the most prevalent valvular disorder in industrialised countries. For patients with severe AS, as well as those with aortic regurgitation who are unsuitable candidates for valve repair, aortic valve replacement (AVR) remains the definitive treatment option [1,2]. Annually, this procedure is performed on approximately 280,000 patients worldwide [2,3]. The last three decades of aortic valve surgery have primarily seen a shift away from the gold standard of median sternotomy (MS) towards less aggressive, minimally invasive surgeries (MIS) [4,5]. Moreover, a decrease in periprocedural mortality has also been observed [4,6]. The two most commonly used MIS approaches are partial upper mini-sternotomy (PUMS) and right anterolateral mini-thoracotomy (RAMT). PUMS involves a shorter incision, typically extending from the top of the sternum down to the right third or fourth intercostal space. RAMT, on the other hand, is a surgical approach without sternum involvement, usually performed through the right second intercostal space anteriorly [4,5,6]. The use of MIS compared to full MS in AVR offers the patient the promise of improved respiratory mechanics, earlier mobilisation and an aesthetically pleasing cosmetic result [4]. Although MIS has been in use in cardiac surgery since the mid-1990s, only a minority of patients undergoing surgery for AS are treated using this approach [7,8].

The implementation of a new surgical technique leads to suboptimal outcomes during the initial stages of its use. Suboptimal results are documented by a learning curve, which characterises the time required to master the new technique and achieve optimal results. The duration and negative impacts of the learning curve period are dependent on the complexity of the new procedure. With the introduction of certain surgical procedures, such as the Ross procedure, the literature indicates up to a five-fold increase in mortality and a more than 20% increase in cardiopulmonary bypass (CPB) time and aortic clamping time [9]. The implementation of endovascular techniques is associated with an increase in mortality, operative time and postoperative complications, including endoleak [10]. The choice of a new surgical approach for myocardial revascularisation also leads to an extension of operative time and an increased risk of perioperative myocardial infarction [11]. The exact impacts of the learning curve on the outcomes of cardiac surgical interventions when introducing new operative approaches are not well established [7,12].

The aim of this study is to retrospectively evaluate the outcomes of AVR via PUMS during the learning curve phase and compare them with those of standard full MS.

## 2. Material and Methods

Minimally invasive methods were introduced at the Department of Heart Surgery of the East Slovak Institute of Cardiovascular Diseases and the Faculty of Medicine at Pavol Jozef Šafárik University in Košice (DHS) in the fourth quarter of 2022. From the outset, a liberal approach was taken in selecting the surgical approach. For patients undergoing isolated AVR, the surgeon could choose either the MS or the PUMS approach after consulting with the patient. All patients undergoing primary AVR due to isolated AS between July 2022 and December 2024 at the DHS were included in the retrospective analysis. Patients with concomitant surgical procedures and those who underwent surgery via the RAMT approach were excluded from the study. Ultimately, 211 patients were included in the cohort. All patients received conventional stented prostheses implanted using individual pledgeted sutures. Neither rapid deployment nor sutureless prostheses were used in any patient. In both surgical approaches, MS and PUMS, cardiopulmonary bypass (CPB) was conducted via central cannulation by means of MS or PUMS; peripheral cannulation was not employed in any patient included in the study.

For all patients, the following preoperative data were recorded: age, sex, ischaemic heart disease (IHD) history, pacemaker implanted before surgery, atrial fibrillation (AF) history (paroxysmal, persistent or permanent), left ventricle ejection fraction (LVEF), indexed aortic valve area (iAVA), aortic valve velocity, body surface area (BSA), body mass index (BMI), diabetes mellitus (DM) history, liver disease history, stroke history and chronic obstructive pulmonary disease (COPD) history. Next, the following perioperative and postoperative data were recorded: primary chosen type of surgical approach (full sternotomy or partial upper mini-sternotomy), conversion to full sternotomy if partial upper mini-sternotomy was chosen, CPB time, aortic clamping time, early revision because of haemorrhage, late revision because of tamponade, wound revision because of deep sternal infection, type of implanted prosthesis (mechanical prosthesis/bioprosthesis), prosthesis diameter, indexed effective orifice area (iEOA) of the implanted valve prosthesis, postoperative atrioventricular block (AVB) with pacemaker implantation needed, persistent postoperative atrial fibrillation (PPOAF), paroxysmal postoperative atrial fibrillation (POAF), duration of stay in an intensive care unit (ICU) and hospitalisation duration. Finally, the following echocardiographic parameters during the follow-up of patients were recorded: median pressure gradient on the prosthesis, peak pressure gradient on the prosthesis, velocity through the prosthesis and any recorded paravalvular leak of the prosthesis.

**Figure 1:**
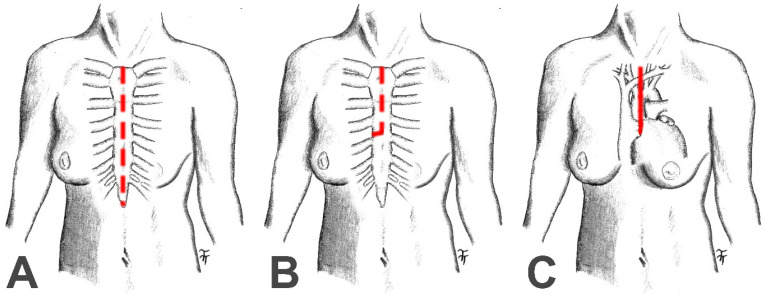
Sketch of the full median sternotomy (**A**), the partial upper mini-sternotomy (**B**) and the relationship of the partial upper mini-sternotomy to the positions of the ascending aorta, aortic valve and right atrial appendage (**C**).

The patients were divided into two groups based on the chosen surgical approach: patients with full MS (n = 119) and patients with PUMS (n = 92) (Figure 1). Patients were then divided into quarterly segments based on the date of the surgery, and the proportional use of full MS and PUMS was evaluated for each quarter. The remaining evaluated data were analysed collectively across all subgroups. Quantitative data are expressed in the statistical analysis as mean ± standard deviation. Qualitative data are expressed as the percentage of positive cases. A two-sample t-test was used to compare the groups of patients with full MS and PUMS (for quantitative parameters), and the chi-square or Fisher’s exact test was used (for qualitative parameters). Patient survival was recorded based on the contractual relationship with the health insurance provider. Survival outcomes in the two patient groups (MS and PUMS) were compared using Kaplan–Meier survival curves and the log-rank test. Last but not least, the change in CPB time and cross-clamp time was assessed during the initial use of the PUMS surgical approach. Patients in the PUMS group were divided into two groups of 46 patients each. The first group included patients undergoing surgery in the initial phase of MIS introduction (Nov. 2022—Nov. 2023). The second group included patients undergoing surgery in the later phase of MIS implementation (Nov. 2023—Dec. 2024). The duration of CPB and aortic clamp time was evaluated between these groups using the Mann–Whitney test. Statistical evaluation was performed using SPSS version 20. Parameters with *p* < 0.05 were considered statistically significant in every assessment.

## 3. Results

The average age of patients in the group was 67 ± 8.7 years. The cohort included 77 (36.5%) women. Ischaemic heart disease without the need for revascularisation was diagnosed in 36% of patients; 1.4% had a pacemaker implanted, and 2.4% had atrial fibrillation diagnosed. The average left ventricular ejection fraction (LVEF) for the entire group was 55.1 ± 9.6%; the average velocity across the aortic valve was 4.6 ± 0.7 m/s, and the indexed aortic valve area (iAVA) was 0.4 ± 0.1 cm^2^/m^2^. The average body surface area (BSA) was 2.0 ± 0.2 m^2^ and the average BMI was 30.6 ± 5.7 kg/m^2^. Diabetes mellitus, liver disease, history of stroke and chronic obstructive pulmonary disease were present in 27%, 12%, 6% and 15% of patients, respectively. The primary approach of upper partial mini-sternotomy was chosen for 94 (44.5%) patients, with conversion to full sternotomy necessary in two cases. One patient experienced a right ventricular injury during the drain placement, and anatomical relations precluded adequate visualisation of the valve from the upper partial mini-thoracotomy approach in another. The average cardiopulmonary bypass time for the entire group was 88 ± 22 min, and the aortic clamp time was 71 ± 17 min. Early revision for bleeding was required in 1.4% of patients and late revision for pericardial tamponade in 6.2%. A biological valve was implanted in 204 (96.7%) patients. The average size of the implanted prosthesis was on average 22.7 ± 1.7 mm, and the indexed effective orifice area (iEOA) was 0.9 ± 0.1 cm^2^/m^2^. The length of stay in the intensive care unit was 4.3 ± 4.4 days, and total hospitalisation was 10.6 ± 5.5 days. Postoperatively, a new atrioventricular block requiring pacemaker implantation was diagnosed in 3.3% of patients, new onset PPOAF was diagnosed in 3.8%, and POAF was diagnosed in 34.1% of patients. During follow-up, the median aortic gradient measured was 13.5 ± 4.5 mmHg, the peak aortic gradient was 24.7 ± 8.7 mmHg and velocity at the site of the aortic prosthesis was 2.4 ± 0.4 m/s. Paravalvular leak was noted in 8.7% of patients, with no reoperation necessary due to its significance. Reoperation was documented in only one patient due to prosthetic infective endocarditis 65 days post-primary surgery.

**Figure 2:**
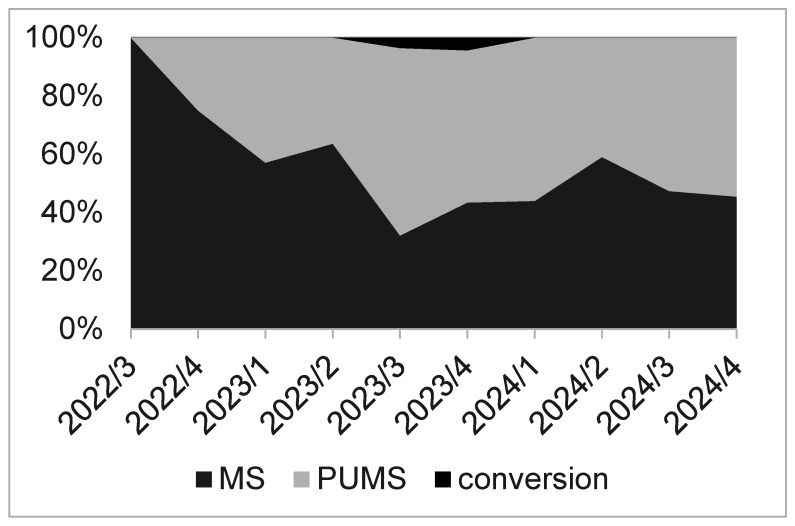
The ratio of utilisation of full sternotomy (MS) and partial upper mini-sternotomy (PUMS) in the quarters since the implementation of the methodology at the department.

Out of a total of 211 patients, the PUMS approach was primarily chosen for 92 patients. A liberal approach to the selection of the surgical approach was adopted at the department, allowing the surgeon to decide which of the two surgical approaches to choose for a patient. Nine months after the introduction of the PUMS approach, it was being used in more than half of the patients undergoing isolated AVR for AS. Subsequently, PUMS was consistently chosen for 40–60% of patients (Figure 2).

**Figure 3:**
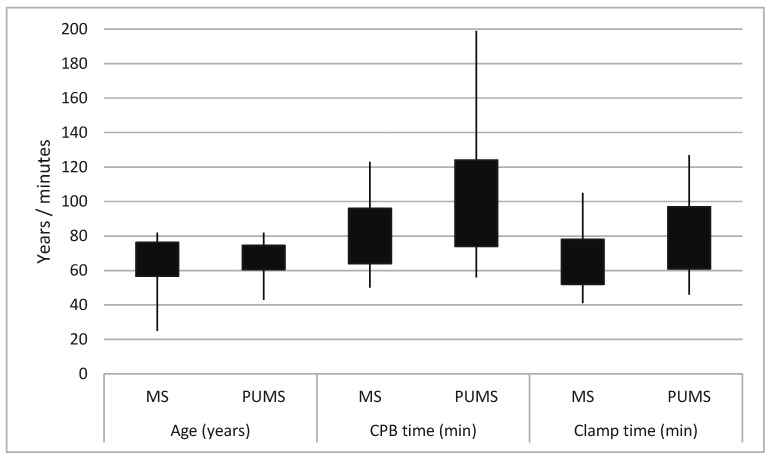
A graph depicting the parameters with statistically significant differences was documented (CPB—cardiopulmonary bypass; MS—medial sternotomy; PUMS—partial upper mini-sternotomy).

**Figure 4:**
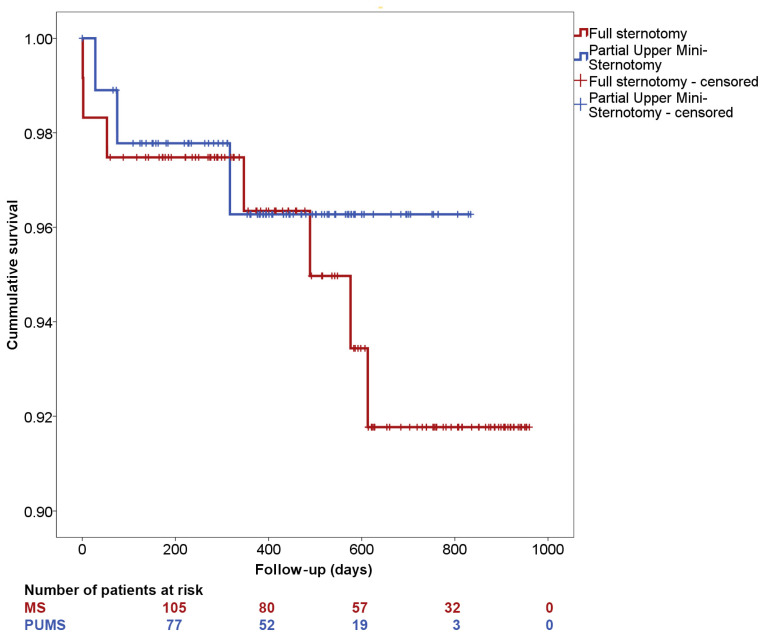
Kaplan–Meier analysis of postoperative survival (log-rank test: *p* = 0.590) (MS—medial sternotomy/full sternotomy; PUMS—partial upper mini-sternotomy).

**Figure 5:**
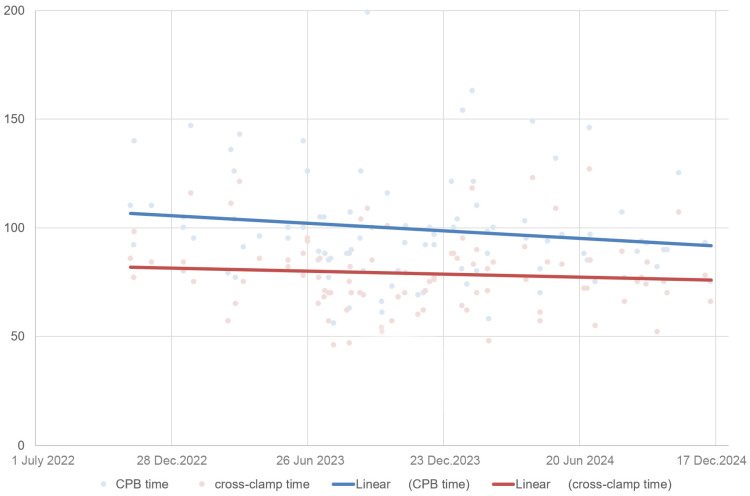
The trend in the CPB time and cross-clamp time during the learning curve phase for implementing the PUMS approach for AVR (AVR—aortic valve replacement; CPB—cardiopulmonary bypass; PUMS—partial upper mini-sternotomy).

A comparison of the preoperative parameters of both groups revealed a difference only in patient age. Patients who underwent surgery via PUMS were on average one year older with statistical significance at the time of the surgery (*p* = 0.010) (Table 1, Figure 3). A comparison of the perioperative parameters indicated a statistically significant longer duration of CPB and aortic cross-clamp time in the PUMS group by 19 and 14 min, respectively (*p* = 0.002 and *p* = 0.024, respectively) (Table 2, Figure 3). Deep sternal infection was recorded in one patient with full MS and in one patient with PUMS. In the patient with PUMS, the deep sternal infection was noted in the case where conversion to full MS was necessary. No other differences were noted between the groups when comparing the sizes of implanted valves, length of hospitalisation, postoperative complications or echocardiographic parameters during patient follow-up. The Kaplan–Meier survival analysis and the log-rank test showed no difference in patient survival between the different surgical approaches (Figure 4). The study of CPB and aortic clamp duration documented a slight, statistically insignificant reduction in CPB duration (98 ± 23 min in the late phase vs. 101 ± 27 min in the initial phase of MIS introduction, *p* = 0.601). No change in aortic clamp duration was observed (79 ± 17 min in the late phase vs. 79 ± 18 min in the initial phase of MIS introduction, *p* = 0.935). The change in the duration of CPB and cross-clamp time during the implementation of PUMS is shown in Figure 5.

## 4. Discussion

One method used in the implementation of a new therapeutic strategy is the proper selection of patients. The technique studied may thus be favoured over the gold standard during the learning curve phase. When generalising the procedure to non-selective patients, this can lead to a deterioration of the observed parameters. A smaller study involving 142 AVR, 55 of which were performed using PUMS, found no differences in the initial parameters of patients operated on using MIS compared to MS [7]. A study focusing on octogenarians also documented a higher average age of patients for whom the surgeon chose MIS. In that study, the choice of approach was also left to the individual surgeon’s discretion [13]. In our group as well, the choice of surgical approach was entirely left to the discretion of the surgeon. Despite this liberal approach, the group with PUMS did not have an advantage based on preoperative parameters.

The duration of CPB and cross-clamp times are frequently evaluated parameters in cardiac surgery, as they largely reflect the complexity of the surgical intervention and the mastery of the surgical procedure. A smaller study involving 55 patients treated with PUMS identified only statistically insignificant differences in CPB and aortic cross-clamp times. In MIS, only a 3 min increase in CPB time and a 9 min increase in aortic cross-clamp time were documented [7]. Another study involving 100 patients operated on with MS and 100 with PUMS corroborated our results by documenting an extension of CPB time by 6.5 min and a cross-clamp time by 8.5 min [12]. The literature also indicates a prolongation of the total operative time when comparing PUMS and MS [14]. A retrospective study focusing on patients with a BMI > 30 kg/m^2^, including 184 patients after propensity score matching, recorded a similar CPB time between MS and PUMS, while the aortic cross-clamp time was significantly shorter with MS [15]. The temporal evolution of CPB time and cross-clamp time in patients operated on using the PUMS approach suggests that during the learning curve phase, there is a more pronounced reduction in CPB time compared to cross-clamp time. When evaluating these surgical approaches after completing the learning curve, we can assume a reduction in the differences in CPB times when comparing MS and PUMS, while the difference in cross-clamp time will persist. Italian authors in their study sought to identify computed tomography parameters responsible for the extension of CPB and cross-clamp times in PUMS. They identified factors such as body surface area > 1.9 m^2^, an enlarged aortic annulus diameter greater than 23 mm, a high calcium score > 2500 Agatston units and a distance between the aorta and sternum greater than 30 mm. The authors reported that these parameters led to a doubling of CPB and cross-clamping times if PUMS was chosen [16]. The duration of CPB and cross-clamp time can be significantly reduced with the use of rapid deployment or sutureless prostheses. The implantation of these prostheses, in comparison to conventional stented valves, offers a notable advantage in shortening operative times [17]. Sutureless prostheses are utilised to simplify the implantation process in both the PUMS and RAMT approaches to aortic valve replacement [18].

According to some authors, one of the benefits of using PUMS is the reduction of intraoperative haemorrhagic complications, which was documented with a statistically significant reduction in the need for transfusion products [7]. Some Czech authors documented a statistically significant lower postoperative blood loss with the use of PUMS [12]. A single-centre study focusing on AVR in octogenarians reported that PUMS is associated with lower drainage output in the first 24 h postoperatively, shorter mechanical ventilation time, a reduced need for blood products and a shorter hospital stay [13]. Even after applying propensity score matching, the literature documents a statistically significant reduction in blood loss during the first 24 postoperative hours and subsequently a reduced need for blood transfusions when choosing PUMS compared to MS [8]. Another study, which focused on comparing MS and PUMS approaches in obese patients, documented significantly lower drainage outputs 48 h post-surgery and a reduced need for blood transfusions in patients operated on using PUMS. Despite not observing a reduction in ventilation time, the authors documented a shortened length of stay in the intensive care unit with the PUMS approach [15]. Other authors, upon comparing 100 patients operated on using PUMS and 100 patients operated on via MS, found no differences in either postoperative blood loss or length of stay in the intensive care unit [14]. Similarly, our data did not record an increased incidence of early or late surgical revisions that could be attributed to increased bleeding with the use of PUMS during the learning curve phase.

Several studies also focused on evaluating short-term postoperative outcomes for PUMS versus MS. One study involving 200 patients reported similar 3-year survival rates. The authors also found no difference in the incidence of the combined endpoint, which included death, stroke and rehospitalisation. However, the study did not address the effect of the learning curve phase [12]. One small single-centre study documented similar survival rates regardless of the choice of surgical approach (PUMS vs. MS), even over a 5-year period [13]. The literature documents similar outcomes for PUMS and MS not only in AVR but also in aortic valve reimplantation [8]. The literature also reports a positive effect of using PUMS on the quality of life of patients after AVR, utilising data comparing quality of life (QoL). In one group of 115 patients, significantly fewer problems with mobility, pain and daily activities were documented with PUMS compared to MS [19].

The implementation of a new surgical technique inevitably leads to the necessity of influencing the efficiency of the operation itself due to the learning curve. The negative impacts of the learning curve are significantly mitigated by the experience of the surgical team and a department with similar, although not identical, techniques used in the past [20,21]. The introduction of PUMS is considered the first stage of MIS in AVR and thus paves the way for RAMT [21]. At the time of this manuscript’s preparation, a programme for the RAMT approach is being introduced, and at the authors’ institution, combined procedures on the aortic valve and ascending aorta are also being performed via PUMS.

## 5. Conclusions

The prolongation of CPB time and cross-clamp times during the learning curve phase of implementing the PUMS method compared to MS did not negatively affect early surgical outcomes and short-term patient survival. Minimising the surgical approach, even during the learning curve phase, did not lead to a deterioration in the parameters of the implanted valve prostheses. Furthermore, according to the literature and presented data, we can assume that after completing the learning curve, there will be a reduction in CPB time for the PUMS approach. The recorded data suggest that for AVR, the PUMS is a method offering at least a similar surgical outcome.

## Figures and Tables

**Table 1:** Preoperative parameters comparison.

	Full Median Sternotomy (n = 119)	Partial Upper Mini-Sternotomy (n = 92)	*p*
Age	66.5 ± 9.8	67.5 ± 7.0	0.010
Women	37%	36%	0.492
IHD history	40%	30%	0.090
Pacemaker preoperatively	3%	0%	0.177
AF	3%	1%	0.275
LVEF	54 ± 10%	58 ± 9%	0.704
iAVA	0.4 ± 0.1 cm^2^/m^2^	0.4 ± 0.1 cm^2^/m^2^	0.825
Velocity	4.6 ± 0.7m/s	4.7 ± 0.7 m/s	0.868
BSA	2 ± 0.2 m^2^	2.0 ± 0.2 m^2^	0.386
BMI	30.7 ± 5.9	30.5 ± 5.5	0.539
Diabetes mellitus	28%	26%	0.457
Liver disease	13%	12%	0.530
Stroke history	7%	4%	0.335
COPD	13%	17%	0.218

**Table 2:** Surgery, hospitalisation and follow-up parameters comparison.

	Full Median Sternotomy (n = 119)	Partial Upper Mini-Sternotomy (n = 92)	*p*
Surgery:			
CPB time	80 ± 16 min	99 ± 25 min	0.002
Clamp time	65 ± 13 min	79 ± 18 min	0.024
Conversion to MS	-	2%	-
Early revisions	1%	2%	0.404
Late revisions	8%	3%	0.103
Deep sternal infections	1%	1%	0.683
Any revisions	10%	5%	0.165
Bioprosthesis	96%	98%	0.341
Prosthesis diameter	22.7 ± 1.8 mm	22.7 ± 1.5 mm	0.060
iEOA	0.88 ± 0.12 cm^2^/m^2^	0.9 ± 0.1 cm^2^/m^2^	0.298
Hospitalisation:			
Pacemaker implantation (AVB)	5%	1%	0.112
PPOAF	4%	3%	0.509
POAF	36%	32%	0.290
ICU	5.5 ± 5.2 days	4.1 ± 3.3 days	0.191
Hospitalisation	10.8 ± 6.2 days	10.3 ± 4.5 days	0.110
Follow-up:			
Medial gradient	13 ± 5 mmHg	14 ± 4 mmHg	0.720
Peak gradient	24 ± 9 mmHg	25 ± 8 mmHg	0.557
Velocity	2.4 ± 0.4 m/s	2.5 ± 0.4 m/s	0.913
Paravalvular leak	7%	11%	0.546

## Data Availability

All important data are contained in the text of the manuscript. Raw data are available to the journal editor if requested.

## Abbreviations and Acronyms

AS	aortic stenosis
AVB	atrioventricular block
AVR	aortic valve replacement
BMI	body mass index
BSA	body surface area
CAPD	chronic obstructive pulmonary disease
CPB	cardiopulmonary bypass
DHS	Department of Heart Surgery of the East Slovak Institute of Cardiovascular Diseases and the Faculty of Medicine at Pavol Jozef Šafárik University in Košice
DM	diabetes mellitus
iAVA	indexed aortic valve area
ICU	intensive care unit
iEOA	indexed effective orifice area
IHD	ischaemic heart disease
LVEF	left ventricle ejection fraction
MS	median sternotomy
POAF	paroxysmal postoperative atrial fibrillation
PPOAF	persistent postoperative atrial fibrillation
PUMS	partial upper ministernotomy
RAMT	right anterolateral minithoracotomy
